# Cellular Effects and Regulated Protein Expression of MCF-7 Breast Cancer Cells Following Exposure to PAH Derivative 3-Hydroxybenz[a]anthracene

**DOI:** 10.3390/toxics14030222

**Published:** 2026-03-04

**Authors:** Xiao Kang, Wenting Song, Xueyan Li, Yuyan Yang, Xinke Wu

**Affiliations:** 1 School of Medicine, Henan Polytechnic University, Jiaozuo 454000, China; 212322020002@home.hpu.edu.cn (X.K.); lixueyan2026@163.com (X.L.); 212523020006@home.hpu.edu.cn (Y.Y.); 212423020012@home.hpu.edu.cn (X.W.); 2 Jiaozuo Key Laboratory for Huaiyao Comprehensive Development, Henan Polytechnic University, Jiaozuo 454000, China

**Keywords:** PAH derivatives, 3-Hydroxybenz[a]anthracene, breast cancer cells, estrogenic activity, protein regulation

## Abstract

Breast cancer is the most common malignant tumor among women worldwide, and its occurrence is closely associated with long-term exposure to environmental pollutants. Polycyclic aromatic hydrocarbons (PAHs) are a class of persistent organic pollutants widely present in the living environment. Epidemiological studies indicate that exposure to PAHs increases the risk of breast cancer. PAH derivatives exhibit stronger toxicity or endocrine-disrupting activity than their parent compounds; however, research on their specific effects and mechanisms in breast cancer cells remains limited. For this purpose, this study selected 3-Hydroxybenz[a]anthracene, a PAH derivative with potential estrogenic activity, as the target compound. Using the estrogen receptor-positive breast cancer cell line MCF-7 as the model, we performed EdU staining, colony formation assays, scratch healing assays, Transwell invasion assays, and apoptosis assays and preliminarily examined changes in relevant signaling proteins via Western blot. Results indicate that 3-Hydroxybenz[a]anthracene promotes proliferation and migration in MCF-7 cells while inhibiting apoptosis under certain conditions, but it has no effect on cell invasion. Mechanistically, it upregulates key proteins including AKT, c-Myc, E-Cadherin, Vimentin, MMP2, MMP9 and Bcl-2 while downregulating p-AKT expression. This study confirms through in vitro experiments that 3-Hydroxybenz[a]anthracene exhibits estrogen-like effects and modulates malignant behavior in breast cancer cells by regulating relevant signaling pathways. These findings provide experimental evidence for further evaluating the potential role of this environmental contaminant in breast cancer initiation and progression.

## 1. Introduction

Cancer represents one of the most pressing societal, public health, and economic challenges of the 21st century [1]. Among women, breast cancer is the most frequently diagnosed malignancy worldwide. Data reported by the International Agency for Research on Cancer (IARC) in 2022 indicate that breast cancer constitutes approximately 11.7% of all newly diagnosed cancer cases in women and is responsible for about 6.9% of global cancer-related mortality [2]. Breast cancer is characterized by pronounced molecular heterogeneity and is broadly classified into four major subtypes: Luminal A, Luminal B, human epidermal growth factor receptor 2 (HER-2)-enriched, and triple-negative breast cancer [3]. Among these molecular categories, the Luminal A and Luminal B subtypes constitute the majority of diagnosed cases [4]. Estrogen receptor-positive breast cancers are predominantly managed using endocrine-based therapeutic strategies, including selective estrogen receptor modulators and aromatase inhibitors [5]. In addition, treatment regimens frequently incorporate targeted approaches such as tyrosine kinase inhibitors or antibody-drug conjugates, alongside radiotherapy and axillary surgical interventions, as integral components of comprehensive disease management [6,7]. Despite significant progress in therapeutic approaches, the global incidence of breast cancer continues to increase, with an emerging shift toward earlier age at diagnosis [8]. Epidemiological evidence suggests that chronic exposure to environmental contaminants in air, water, and soil may substantially contribute to elevated breast cancer risk [9]. Consequently, elucidating the mechanistic pathways through which environmental pollutants drive breast cancer initiation and progression is essential for the development of more effective prevention and disease control strategies.

Polycyclic aromatic hydrocarbons (PAHs) constitute a major class of persistent organic pollutants composed of two or more fused aromatic rings and are ubiquitously distributed across environmental matrices [10]. Their origins are broadly classified as anthropogenic, with human-related activities accounting for the dominant contribution to environmental PAH emissions. Major anthropogenic sources of PAHs include industrial effluents, vehicular emissions, energy generation processes, waste incineration, biomass burning, and residential heating and cooking activities [11,12,13,14]. PAHs have been detected in various environmental media, including air, soil, and water, and their concentrations vary significantly depending on region and pollution sources. For instance, total PAH concentrations in air can range from 0.03 to 0.60 ng/m^3^ in rural areas to as high as 12,300 ng/m^3^ in industrial regions [15]. Studies have shown that PAH concentrations in air reach relatively high levels in industrial/urban areas, while PAH levels in soil also differ significantly across various functional zones [16]. Humans are exposed to PAHs primarily through three routes: inhalation, ingestion, and dermal contact [17]. These compounds are well documented to exhibit genotoxic, mutagenic, and carcinogenic properties [18,19], and chronic exposure has been associated with an increased risk of multiple malignancies, including colorectal and lung cancers [20,21]. In recent years, advances in analytical methodologies and the expanding scope of environmental health research have brought increasing attention to oxidized, nitrated, and hydroxylated PAH derivatives formed through environmental transformation processes or biological metabolism. Although these derivatives are typically present at lower environmental concentrations than their parent PAHs, they often display markedly enhanced toxicity, mutagenicity, and carcinogenicity. This increased hazard is attributed to mechanisms such as direct induction of DNA damage and the generation of reactive oxygen species (ROS), which can occur independently of metabolic activation [22,23,24,25].

Epidemiological evidence demonstrates that chronic exposure to PAHs and their derivatives is associated with an elevated risk of breast cancer at the population level and correlates with adverse clinical outcomes and poorer prognosis among affected patients [26,27,28]. Several hydroxylated PAH derivatives have been reported to exert estrogen-like biological activity, attributable to their structural resemblance to the endogenous hormone 17β-estradiol (E_2_) [29]. However, the molecular mechanisms by which hydroxylated PAH derivatives modulate malignant phenotypes in breast cancer cells via estrogen-dependent signaling pathways remain inadequately defined. Among these compounds, 3-Hydroxybenz[a]anthracene (3-OH-B[a]A) is both a widely distributed atmospheric contaminant [30] and a major hydroxylated metabolite of benz[a]anthracene, a representative parent PAH detected in human urine [31]. Competitive binding assays and yeast two-hybrid systems expressing the human estrogen receptor (hER) have demonstrated that 3-OH-B[a]A is capable of directly interacting with hER and exhibits estrogenic activity [32]. However, its estrogen-mimetic effects on breast cancer cells and the associated toxicological mechanisms have yet to be systematically characterized. Accordingly, the estrogen receptor-positive breast cancer cell line MCF-7 was employed as an in vitro model to evaluate the effects of 3-hydroxy-B (a)A systematically on key malignant phenotypes, including cell proliferation, migration, invasion and apoptosis, and to elucidate the underlying molecular mechanisms governing these responses. This study provides experimental evidence for assessing the estrogen-mimetic potential of PAH derivatives and contributes to improved environmental risk assessment and prevention strategies for breast cancer. In addition, it offers mechanistic insights that may facilitate the identification of relevant molecular targets and inform the development of targeted therapeutic interventions.

## 2. Materials and Methods

### 2.1. Materials

3-OH-B[a]A was purchased from Shanghai Aladdin Bio-Chem Technology Co., Ltd. (Shanghai, China). 17β-estradiol (E_2_) was obtained from Shandong Sikejie Biotechnology Co., Ltd. (Jinan China). The stock solutions were prepared by dissolving the chemical in dimethylsulfoxide (DMSO, MP Biomedicals, Irvine, CA, USA) and kept at 4 °C in darkness. Activated charcoal-treated fetal bovine serum was purchased from Beijing Zoman Biotechnology Co., Ltd. (Beijing, China). One-Step TUNEL Apoptosis Detection Kit were obtained from Abbkine Scientific Co., Ltd. (Wuhan, China). Cell-Light EdU Apollo567 in vitro kit was purchased from Guangzhou Ribo Bio Co., Ltd. (Guangzhou, China). Triton X-100 and Glycine were purchased from Macklin Corporation (Jinan, China). Crystal violet saining solution was purchased from Shanghai Beyotime Biotechnology Co., Ltd. (Shanghai, China). Paraformaldehyde was obtained from Wuhan Servicebio Technology Co., Ltd. (Wuhan, China). Matrigel matrix was purchased from ABW (Shanghai, China).

### 2.2. Cell Line, Culture and Working Solutions

Human breast cancer cell MCF-7 was used as the in vitro assay model in this paper. MCF-7 cells were cultured in DMEM (Sikejie, Jinan, China) containing 10% fetal bovine serum (FBS, Abbkine, Wuhan, China), 100 U/mL penicillin, and 100 µg/mL streptomycin (Sikejie, Jinan, China). The incubations were kept in a humidified atmosphere of 5% CO_2_ at 37 °C. MCF-7 cells were used for experiments after at least two passages following thawing to ensure recovery and stable growth.

The 3-OH-B[a]A mother liquor was diluted into 0.4 μM and 2 μM working solution with complete culture medium; the E_2_ mother solution was diluted into 0.5 nM and 5 nM working solution, and the final concentration of DMSO is ≤0.1%.

### 2.3. Antibobies

Primary antibodies used were: rabbit monoclonal to β-Actin (Beyotime, Shanghai, China, Cat # AF5003; 1:1000 dilution); mouse monoclonal to AKT (Proteintech, Wuhan, China, Cat # 60203-2-Ig; 1:5000 dilution); rabbit monoclonal to p-AKT (Ser473) (Beyotime, Shanghai, China, Cat # AF1546; 1:1000 dilution); rabbit polyclonal to c-Myc (Beyotime, Shanghai, China, Cat # AF6513; 1:1000 dilution); mouse monoclonal to E-Cadherin (Beyotime, Shanghai, China, Cat # AF0138; 1:1000 dilution); mouse monoclonal to Vimentin (Beyotime, Shanghai, China, Cat # AF0318; 1:1000 dilution)rabbit polyclonal to MMP2 (Proteintech, Wuhan, China, Cat # 10373-2-AP; 1:1000 dilution); rabbit polyclonal to MMP9 (Beyotime, Shanghai, China, Cat # AF5234; 1:1000 dilution); rabbit monoclonal to Bax (Beyotime, Shanghai, China, Cat # AF1270; 1:1000 dilution); rabbit monoclonal to Bcl-2 (Proteintech, Wuhan, China, Cat # 80313-1-RR; 1:5000 dilution). Secondary antibodies used were: HRP-labeled Goat Anti-Rabbit IgG(H+L) (Beyotime, Shanghai, China, Cat # A0208; 1:1000 dilution); HRP-labeled Goat Anti-Mouse IgG(H+L) (Beyotime, Shanghai, China, Cat # A0216; 1:1000 dilution).

### 2.4. EdU Cell Proliferation Detection

**Figure 1:**
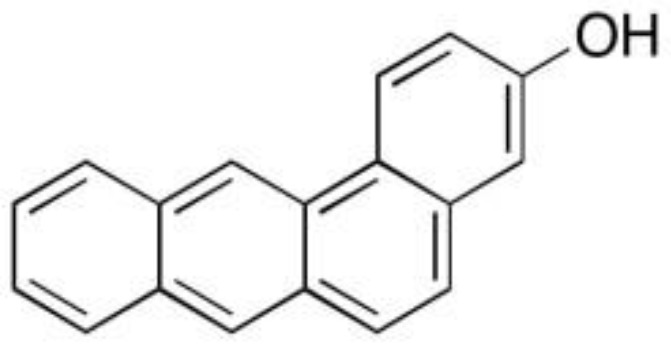
3-OH-B[a]A molecular structure.

The effect of 3-OH-B[a]A (Figure 1) on the proliferation of breast cancer cells was evaluated using an EdU Apollo567 Cell Fluorescence in vitro kit. A stock solution of 3-OH-B[a]A was diluted to working concentrations of 0.4 and 2 μM in a DMEM medium supplemented with 5% activated charcoal-treated fetal bovine serum and 1% penicillin-streptomycin (double antibody). An E_2_ stock solution was diluted to a 0.5 nM working concentration using the same medium, with the final DMSO concentration maintained at ≤0.1% in all groups. MCF-7 cells were pre-cultured in a DMEM medium containing 5% charcoal-stripped FBS and 1% double antibody for 24 h, then seeded into 12-well plates at a density of 1.0 × 10^5^ cells/well and incubated in a CO_2_ incubator. After 24 h of cell adhesion, the medium was replaced with fresh medium containing the aforementioned working solutions; at 68 h post-exposure, the EdU reagent was added, and the cells were further incubated for 4 h, followed by sequential procedures of cell fixation, Apollo staining, and DNA staining.

### 2.5. Colony Formation Assay

MCF-7 cells were seeded into 6-well plates at a density of 800 cells/well and incubated in the CO_2_ incubator; after 24 h of culturing to allow cell adhesion, the original culture medium was discarded, and then the cells in different groups were treated with culture media containing different working solutions; the drug-containing solutions were replaced every 3 days, and the culturing was continued for 15 consecutive days, followed by washing twice with PBS, fixing with a 4% paraformaldehyde solution, and staining with crystal violet.

### 2.6. Scratch Assay

MCF-7 cells were seeded into 24-well plates at a density of 1.8 × 10^5^ cells/well and cultured in the CO_2_ incubator. After the cells adhered to the plate, the medium was replaced with a 2% DMEM medium for 24 h starvation. A 10 μL pipette tip was used to scratch the cell monolayer vertically from top to bottom in each well; afterward, the medium was discarded, and the cells were washed twice with PBS. Subsequently, the cells in different groups were treated with culturing media containing different working solutions, and the same position of the scratch was imaged under a microscope at 0, 12, 24, 36, and 48 h post-scratch exposure.

### 2.7. Transwell Invasion Assay

Matrigel was mixed with a basic medium at a ratio of 1:15, and the mixture was added to the bottom of the Transwell insert, which was then placed in the CO_2_ incubator to allow the gel to solidify. The Transwell insert was taken out, excess liquid was discarded, and an appropriate cell suspension was prepared using a basic medium containing 5 nM E_2_ and different concentrations of 3-OH-B[a]A; MCF-7 cells were seeded into the Transwell insert at a density of 1.5 × 10^5^ cells/insert, while a 10% DMEM medium was added to the lower chamber, followed by incubation in the CO_2_ incubator for 72 h. After incubation, the 24-well plate was taken out, and the matrigel remaining in the upper layer of the Transwell insert was removed with a cotton swab. The insert was washed twice with PBS, fixed with a 4% paraformaldehyde solution, stained with crystal violet, rinsed with ultrapure water, and then inverted and air-dried.

### 2.8. One-Step TUNEL Assay for Apoptosis Detection

The apoptotic effect of 3-OH-B[a]A on breast cancer cells was evaluated using a one-step TUNEL assay kit. Working solutions of 3-OH-B[a]A (0.4 and 2 μM) and E_2_ (0.5 nM) were prepared by diluting the respective stock solutions with the DMEM medium supplemented with 5% activated charcoal-treated fetal bovine serum and 1% penicillin/streptomycin, ensuring a final DMSO concentration of ≤0.1%. MCF-7 cells were seeded in 96-well plates at a density of 1.0 × 10^4^ cells/well and cultured in the same medium for 24 h in the CO_2_ incubator to allow attachment. After 24 h, the medium was replaced with treatment media containing the respective working solutions. Following 72 h of exposure, the cells were fixed and subjected to TUNEL staining and DAPI counterstaining.

### 2.9. Western Blot

For the detection of AKT, p-AKT, c-Myc, Bax, and Bcl-2 proteins, MCF-7 cells were cultured for 24 h in the DMEM medium supplemented with 5% activated charcoal-treated fetal bovine serum and 1% double-antibody. MCF-7 cells were seeded at a density of 5.0 × 10^5^ cells/well in 6-well plates and incubated in the CO_2_ incubator. After 24 h of cell attachment, the 3-OH-B[a]A stock solution was diluted to 0.4 and 2 μM working solutions in the DMEM medium supplemented with 5% activated charcoal-treated fetal bovine serum and 1% dual antibodies; the E_2_ stock solution was diluted to 0.5 and 5 nM working solutions. The final DMSO concentration was ≤0.1%. Media containing different working solutions were added. For the detection of E-Cadherin, Vimentin, MMP2, and MMP9 proteins, MCF-7 cells were seeded at 5.0 × 10^5^ cells/well in 6-well plates and cultured in the CO_2_ incubator. After 24 h of cell attachment, the cells were starved for 24 h in a DMEM medium supplemented with 2% FBS and 1% double antibody. The 3-OH-B[a]A stock solution was diluted into 0.4 and 2 μM working solutions in the DMEM medium containing 2% FBS and 1% double antibody. The E_2_ stock solution was diluted to 0.5 and 5 nM working solutions, ensuring a final DMSO concentration of ≤0.1%. The respective working solutions were added to the culture medium. After 72 h exposure, cells were lysed on ice using a cell lysis buffer containing protease and phosphatase inhibitors to collect total cellular proteins. Protein concentrations were measured using the BCA assay. Proteins were separated using 8% or 12% SDS-PAGE gels prepared from a 30% acrylamide/bis-acrylamide stock solution (29:1). The gels were cast with 1.0 mm thickness. A pre-stained protein molecular weight marker (10–200 kDa) was used to estimate target protein sizes. Electrophoresis was initially performed at 80 V, 400 mA for 40 min, and then continued at 120 V, 400 mA until the protein samples reached the bottom of the gel. After electrophoresis, proteins were transferred to an NC membrane at 250 mA for 90 min under ice-bath conditions. The membrane was blocked with 5% skim milk powder or 5% BSA for 1.5 h, then it was incubated with primary antibody overnight at 4 °C, washed four times with 1 × TBST (10 min/wash), incubated with secondary antibody for 1.5 h, washed four times with 1 × TBST (10 min/wash), and finally visualized using enhanced chemiluminescence.

### 2.10. Experimental Design Overview

The experiments in this study were designed to systematically investigate the cellular and molecular effects of 3-OH-B[a]A on MCF 7 breast cancer cells, following a logical progression from cellular phenotypes to molecular mechanisms. Specifically, cell proliferation was first assessed using EdU incorporation and colony formation assays. Subsequently, cell migration and invasion capabilities were evaluated via wound healing and Transwell invasion assays. Apoptosis was then examined using TUNEL staining. Finally, the underlying molecular mechanisms were explored by Western blot analysis, focusing on key proteins associated with proliferation, migration, invasion, and apoptosis (2–3 core proteins per aspect). This sequential order, from functional phenotypes to mechanistic exploration, ensures logical coherence and mutual validation among experimental results, aligning with the core objectives of this study.

### 2.11. Statistical Analysis

One-way ANOVA was used for the analysis using GraphPad Prism 9.5 software. Data is expressed as mean ± SD. All the experiments were carried out in triplicate to ensure reproducibility.

## 3. Results

### 3.1. Proliferative Effects of 3-Hydroxybenz[a]anthracene in MCF-7 Cells

**Figure 2:**
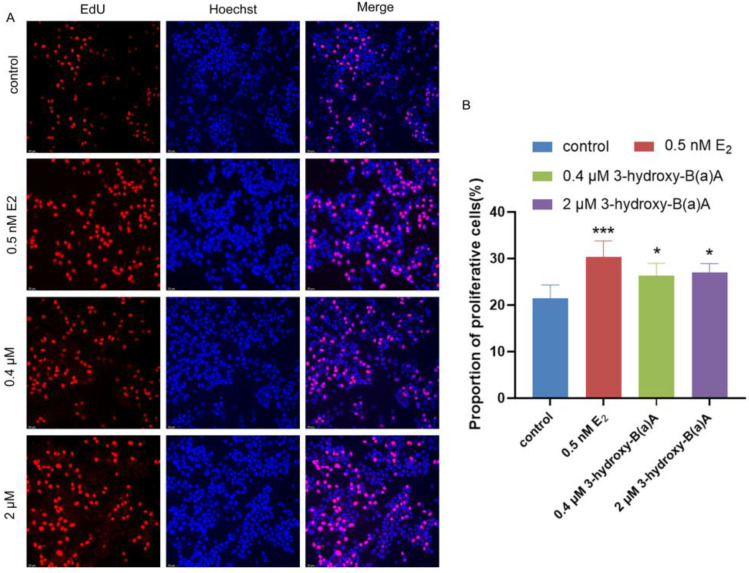
Effects of 3-OH-B[a]A and E_2_ on MCF-7 cell proliferation after 72 h of exposure (*n* = 3). (**A**) Representative fluorescence images showing MCF-7 cell proliferation following treatment with 3-OH-B[a]A and E_2_ for 72 h. (**B**) Quantitative comparison of the proportion of proliferative cells derived from panel (**A**). Data are presented as mean ± SD. * *p* < 0.05 and *** *p* < 0.001 versus the vehicle control.

Following exposure of MCF-7 cells to varying concentrations of 3-OH-B[a]A and 17β-estradiol (E_2_) for 72 h, cell proliferation was assessed, as shown in Figure 2. As shown in Figure 2A, treatment with 0.5 nM E_2_, as well as 0.4 μM and 2 μM 3-OH-B[a]A, resulted in a significant increase in the proportion of EdU-positive cells compared with the control group. Consistently, quantitative analysis presented in Figure 2B demonstrated that the proportion of proliferative cells was significantly elevated in all treatment groups relative to the control (*p* < 0.05 or *p* < 0.001). The proportion of proliferative cells was 30.30% in the 0.5 nM E_2_ group, 26.33% in the 0.4 μM 3-OH-B[a]A group, and 26.99% in the 2 μM 3-OH-B[a]A group. Among the treatment conditions, the 0.5 nM E_2_ positive control displayed the highest proportion of proliferative cells (30.30%). In comparison, exposure to 2 μM 3-OH-B[a]A resulted in a modestly greater proliferative response than the 0.4 μM treatment. Collectively, these findings demonstrate that 3-OH-B[a]A at both 0.4 μM and 2 μM concentrations significantly enhances the proliferative capacity of MCF-7 cells.

### 3.2. Colony-Promoting Effects of 3-Hydroxybenz[a]anthracene in MCF-7 Cells

**Figure 3:**
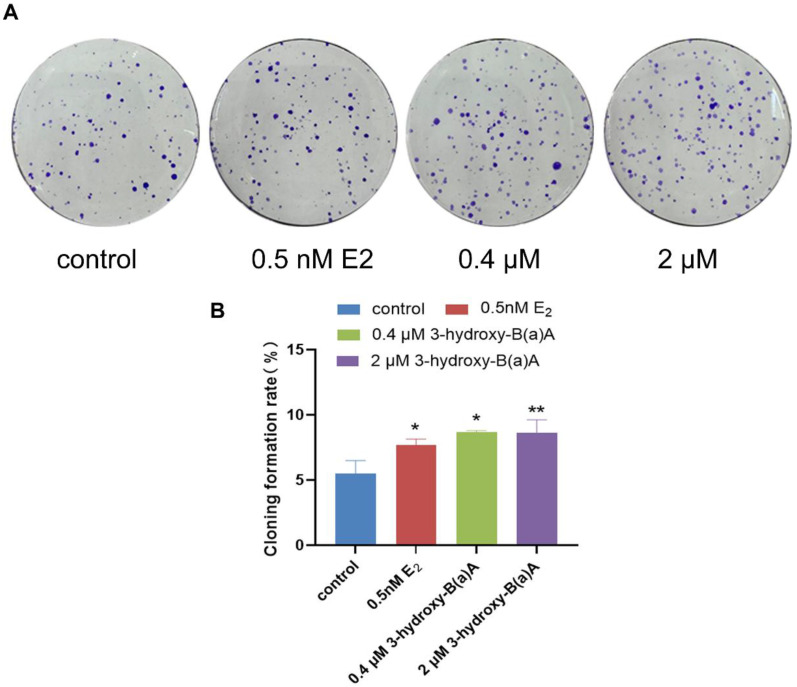
Effects of 3-OH-B[a]A and E_2_ on colony formation of MCF-7 cells after 15 days of exposure (*n* = 3). (**A**) Representative images of MCF-7 cell colonies following treatment with 3-OH-B[a]A and E_2_ for 15 days. (**B**) Quantitative analysis of colony formation rate derived from panel (**A**). Data are presented as mean ± SD. * *p* < 0.05 and ** *p* < 0.01 versus the vehicle control.

The effects of 72 h exposure to varying concentrations of 3-OH-B[a]A and E_2_ on the clonogenic potential of MCF-7 cells are presented in Figure 3. As illustrated in Figure 3A, treatment with 0.5 nM E_2_, as well as 0.4 μM, and 2 μM 3-OH-B[a]A resulted in a marked increase in the number of colonies formed compared with the control group. Quantitative evaluation shown in Figure 3B further confirmed that colony formation rates were 7.67% in the 0.5 nM E_2_ group, 8.69% in the 0.4 μM 3-OH-B[a]A group, and 8.63% in the 2 μM 3-OH-B[a]A group, all of which were significantly elevated compared with the control group. They exceeded those observed in the 0.5 nM E_2_ positive control group (*p* < 0.05 or *p* < 0.01). The colony-forming efficiencies observed in MCF-7 cells treated with 0.4 μM and 2 μM 3-OH-B[a]A were comparable. Collectively, these findings indicate that exposure to 3-OH-B[a]A at both concentrations significantly enhances the clonogenic potential of MCF-7 cells.

### 3.3. Migratory Effects of 3-Hydroxybenz[a]anthracene in MCF-7 Cells

**Figure 4:**
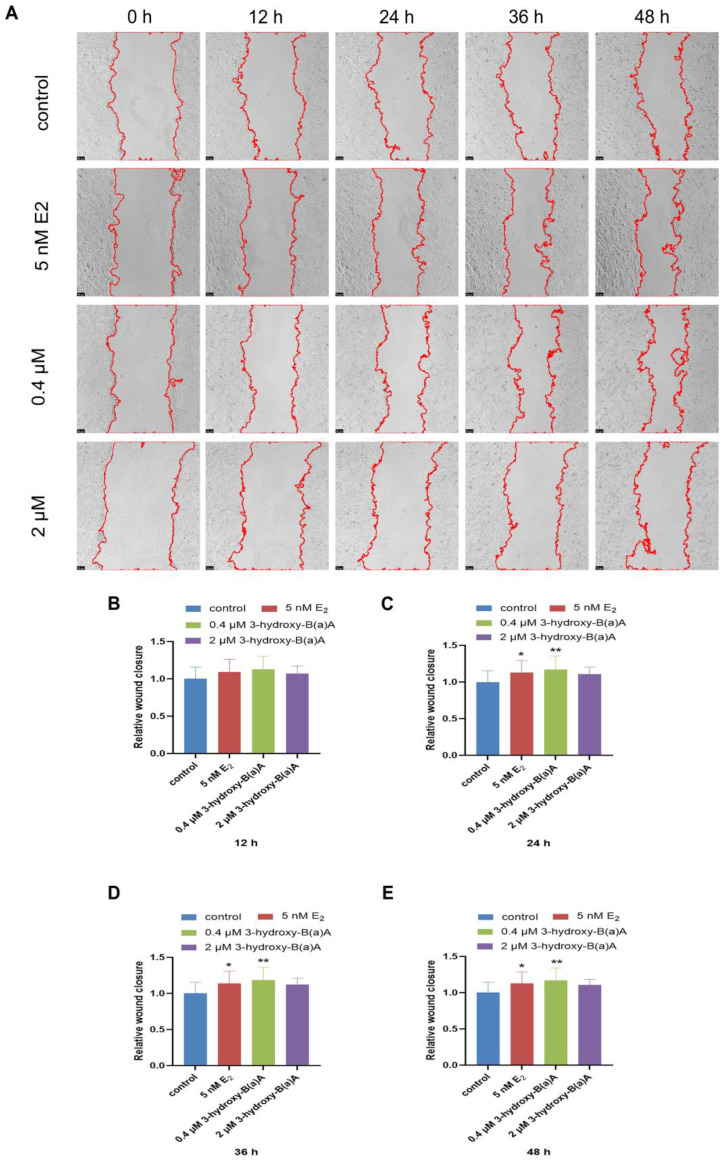
Effects of 3-OH-B[a]A and E_2_ on the migration of MCF-7 cells over 0-48 h (*n* = 3). (**A**) Representative images showing MCF-7 cell migration following treatment with 3-OH-B[a]A and E_2_ for 0, 12, 36, and 48 h. (**B**–**E**) Quantitative comparison of the relative migration area derived from panel (**A**). Data are presented as mean ± SD. * *p* < 0.05 and ** *p* < 0.01 versus the vehicle control.

The migratory behavior of MCF-7 cells following exposure to varying concentrations of 3-OH-B[a]A and E_2_ over 0, 12, 24, 36, and 48 h is presented in Figure 4. As shown in Figure 4C–E, treatment with 5 nM E_2_ and 0.4 μM 3-OH-B[a]A resulted in significantly increased relative migration areas compared with the control group at 24 h, 36 h, and 48 h (*p* < 0.05 or *p* < 0.01), reaching 1.13 and 1.17 (24 h), 1.14 and 1.18 (36 h), and 1.13 and 1.17 (48 h), respectively. The relative migration area in the 0.4 μM 3-OH-B[a]A-treated group peaked at 36 h, whereas no significant alterations in migratory activity were detected in the 2 μM 3-OH-B[a]A group at any assessed time point (remaining at approximately 1.11, similar to the control). Consistent with these findings, Figure 4B shows that no significant differences in relative migration area were observed among any treatment groups following 12 h of exposure (all groups remained at approximately 1.10). Collectively, these findings demonstrate that exposure to 0.4 μM 3-OH-B[a]A markedly enhances the migration capacity of MCF-7 cells, with the maximal effect observed at 36 h.

### 3.4. Invasive Effects of 3-Hydroxybenz[a]anthracene in MCF-7 Cells

**Figure 5:**
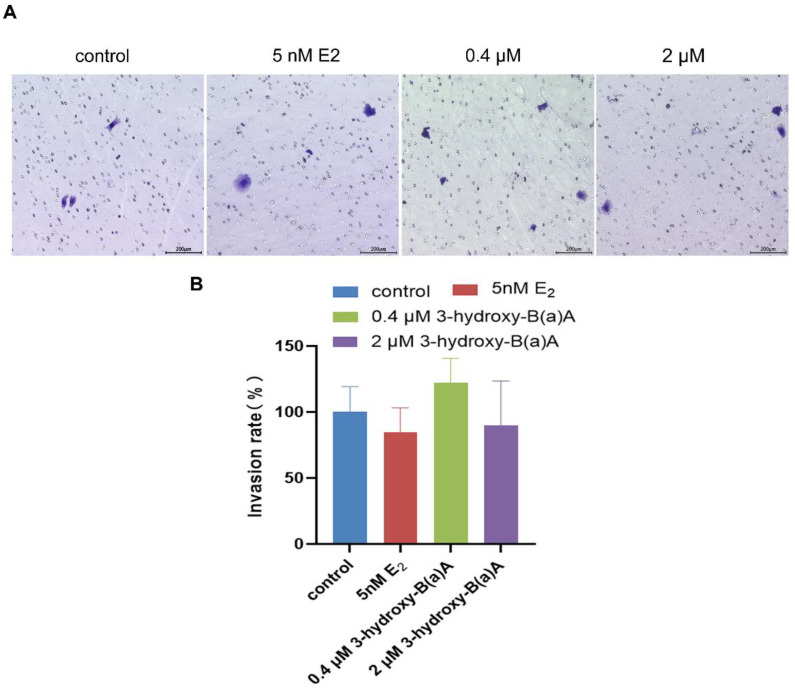
Effects of 3-OH-B[a]A and E_2_ on the invasion of MCF-7 cells after 72 h of exposure (*n* = 3). (**A**) Representative images showing MCF-7 cell invasion following treatment with 3-OH-B[a]A and E_2_ for 72 h. (**B**) Quantitative analysis of the invasion rate derived from panel (**A**). Data are presented as mean ± SD. *p* > 0.05 versus the vehicle control.

Figure 5 illustrates the invasive response of MCF-7 cells following 72 h of exposure to varying concentrations of 3-OH-B[a]A and E_2._ As shown in Figure 5A, none of the tested treatments, including 5 nM E_2_ and 0.4 μM or 2 μM 3-OH-B[a]A, produced a statistically significant increase in the number of cells traversing the transmembrane matrix compared with the untreated control. Quantitative analysis showed that the invasion rates were 100% in the control group, 84.37% in the 5 nM E_2_ group, 121.87% in the 0.4 μM 3-OH-B[a]A group, and 90.00% in the 2 μM 3-OH-B[a]A group. Quantitative analysis confirmed that none of the treatment groups exhibited a statistically significant difference in invasive activity relative to the control group (*p* > 0.05) (Figure 5B). Collectively, these findings demonstrate that, under the present experimental conditions, exposure to 3-OH-B[a]A at concentrations of 0.4 μM and 2 μM does not exert a measurable effect on the invasive potential of MCF-7 cells.

### 3.5. Anti-Apoptotic Effects of 3-Hydroxybenz[a]anthracene in MCF-7 Cells

**Figure 6:**
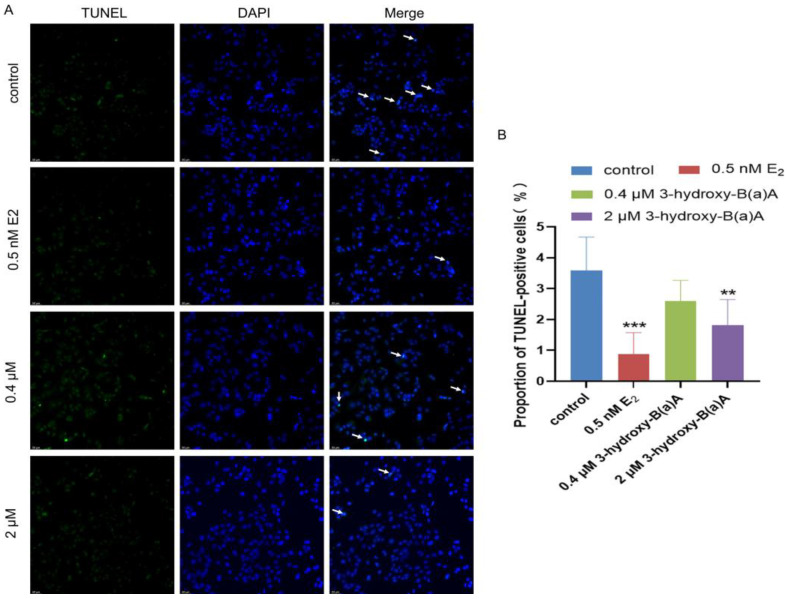
Effects of 3-OH-B[a]A and E_2_ on apoptosis of MCF-7 cells after 72 h of exposure (*n* = 3). (**A**) Representative images showing apoptosis of MCF-7 cells following treatment with 3-OH-B[a]A and E_2_ for 72 h, as assessed by the TUNEL assay. Arrows indicate TUNEL-positive apoptotic cells. (**B**) Quantitative analysis of the proportion of TUNEL-positive cells derived from panel (**A**). Data are presented as mean ± SD. ** *p*< 0.01 and *** *p* < 0.001 versus the vehicle control.

Figure 6 depicts the apoptotic response of MCF-7 cells following 72 h of exposure to varying concentrations of 3-OH-B[a]A and E_2_. As shown in Figure 6A, a marked reduction in the proportion of TUNEL-positive cells was observed in cells treated with 0.5 nM E_2_ and 2 μM 3-OH-B[a]A relative to the untreated control. Quantitative analysis (Figure 6B) revealed that the proportion of apoptotic cells was 3.59% in the control group, 0.87% in the 0.5 nM E_2_ group, 2.60% in the 0.4 μM 3-OH-B[a]A group, and 1.81% in the 2 μM 3-OH-B[a]A group. Treatment with 0.5 nM E_2_ and 2 μM 3-OH-B[a]A resulted in a significant suppression of apoptotic activity (*p* < 0.01 or *p* < 0.001, respectively), with the 0.5 nM E_2_ group exhibiting the lowest proportion of apoptotic cells. In contrast, treatment with 0.4 μM 3-OH-B[a]A did not result in a statistically significant change in apoptosis relative to the control. Collectively, these findings indicate that 2 μM 3-OH-B[a]A exerts a significant anti-apoptotic effect in MCF-7 cells.

### 3.6. 3-Hydroxybenz[a]anthracene Modulates Protein Expression in MCF-7 Cells

**Figure 7:**
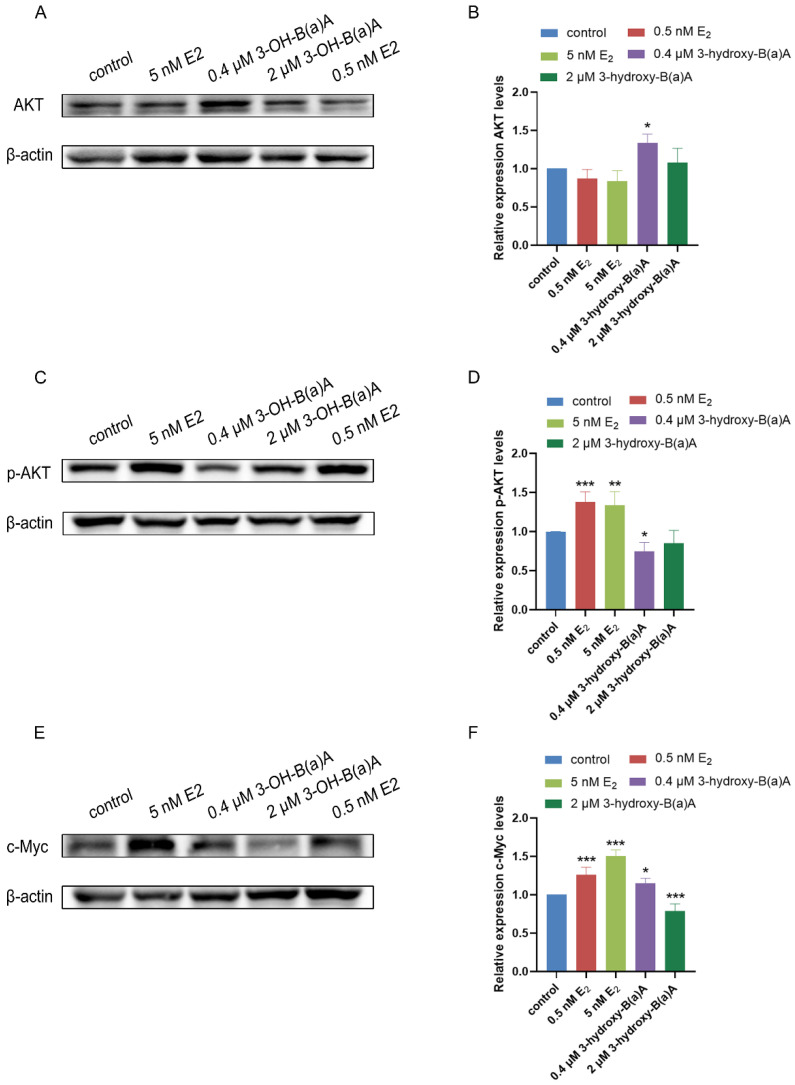
Effect of 3-OH-B[a]A and E_2_ on the expression of AKT, p-AKT, and c-Myc proteins in MCF-7 cells (*n* = 3). (**A**) Representative immunoblot showing AKT expression in MCF-7 cells following treatment with 3-OH-B[a]A and E_2_. (**B**) Quantitative analysis of relative AKT protein expression corresponding to panel (**A**). (**C**) Representative immunoblot showing p-AKT expression. (**D**) Quantitative analysis of relative p-AKT expression corresponding to panel (**C**). (**E**) Representative immunoblot showing c-Myc expression. (**F**) Quantitative analysis of relative c-Myc protein expression corresponding to panel (**E**). Data are presented as mean ± SD. * *p* < 0.05, ** *p* < 0.01, and *** *p* < 0.001 versus the vehicle control.

Exposure of MCF-7 cells to varying concentrations of 3-OH-B[a]A and E_2_ for 72 h resulted in significant changes in the expression levels of selected proteins. Figure 7 presents Western blot analyses evaluating the effects of 3-OH-B[a]A and E_2_ on the expression of AKT, phosphorylated AKT (p-AKT), and c-Myc in MCF-7 cells. As shown in Figure 7A,B, treatment with E_2_ at concentrations of 0.5 nM and 5 nM did not produce any significant alteration in total AKT protein levels relative to the control (relative expression: 0.87 and 0.84, respectively). In contrast, exposure to 0.4 μM 3-OH-B[a]A resulted in a significant upregulation of AKT protein expression (1.34-fold compared with the control), whereas treatment with 2 μM 3-OH-B[a]A produced no statistically significant change (relative expression: 1.08), reflecting the response observed in the E_2_-treated groups. Collectively, these findings demonstrate that 3-OH-B[a]A at 0.4 µM selectively enhances AKT protein expression in human breast cancer MCF-7 cells.

As shown in Figure 7C,D, quantitative analysis revealed that the relative expression levels of p-AKT were 1.00 in the control group, 1.38 in the 0.5 nM E_2_ group, 1.34 in the 5 nM E_2_ group, 0.75 in the 0.4 μM 3-OH-B[a]A group, and 0.85 in the 2 μM 3-OH-B[a]A group, respectively. Treatment with E_2_ at concentrations of 0.5 nM and 5 nM significantly enhanced p-AKT protein expression relative to the control. In contrast, exposure to 0.4 μM 3-OH-B[a]A led to a pronounced reduction in p-AKT levels, whereas the 2 μM treatment did not produce a statistically significant change compared with the control. Collectively, these data demonstrate that 3-OH-B[a]A at 0.4 μM suppresses AKT phosphorylation in human breast cancer MCF-7 cells.

As illustrated in Figure 7E,F, quantitative analysis showed that the relative expression levels of c-Myc were 1.00 in the control group, 1.26 in the 0.5 nM E_2_ group, 1.50 in the 5 nM E_2_ group, 1.15 in the 0.4 μM 3-OH-B[a]A group, and 0.79 in the 2 μM 3-OH-B[a]A group, respectively. Exposure to E_2_ at 0.5 nM and 5 nM significantly increased c-Myc expression relative to the control. Consistent with this trend, treatment with 0.4 μM 3-OH-B[a]A also markedly upregulated c-Myc levels, whereas exposure to 2 μM of the compound resulted in a significant downregulation of c-Myc expression. Taken together, these findings demonstrate a concentration-dependent modulation of c-Myc expression by 3-OH-B[a]A in MCF-7 cells, with 0.4 μM inducing upregulation of c-Myc protein levels, whereas exposure to 2 μM results in a significant downregulation.

**Figure 8:**
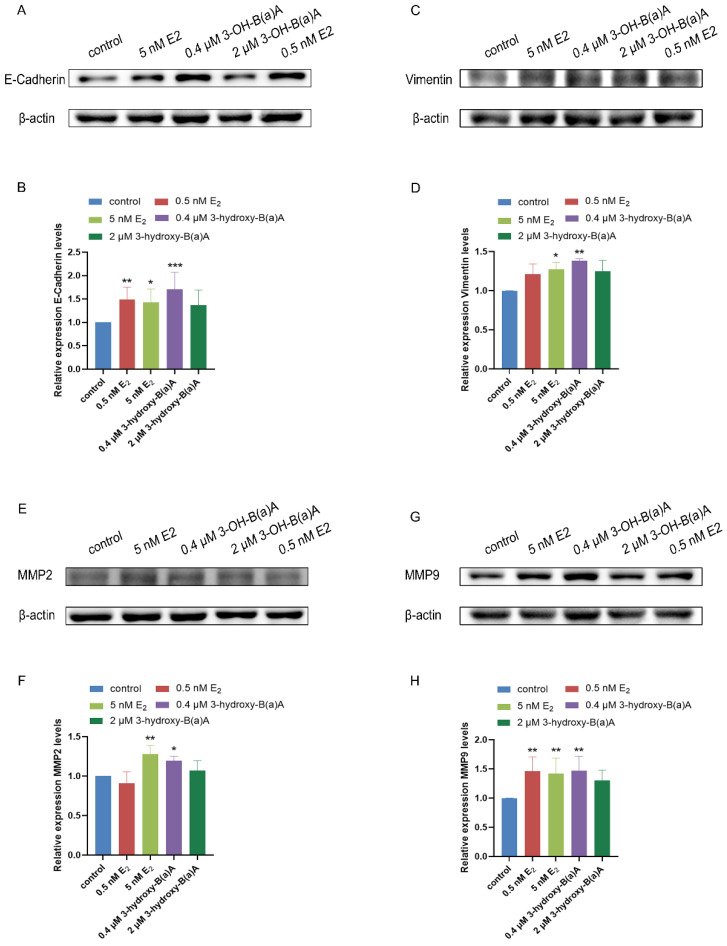
Effects of 3-OH-B[a]A and E_2_ on the expression of E-Cadherin, Vimentin, MMP2, and MMP9 in MCF-7 cells (*n* = 3). (**A**) Representative immunoblot showing E-Cadherin expression following treatment with 3-OH-B[a]A and E_2_. (**B**) Quantitative analysis of relative E-Cadherin protein expression corresponding to panel (**A**). (**C**) Representative immunoblot showing Vimentin expression. (**D**) Quantitative analysis of relative Vimentin protein expression corresponding to panel (**C**). (**E**) Representative immunoblot showing MMP2 expression. (**F**) Quantitative analysis of relative MMP2 protein expression corresponding to panel (**E**). (**G**) Representative immunoblot showing MMP9 expression. (**H**) Quantitative analysis of relative MMP9 protein expression corresponding to panel (**G**). Data are presented as mean ± SD. * *p* < 0.05, ** *p* < 0.01, and *** *p* < 0.001 versus the vehicle control.

Western blot analysis assessing the effects of 3-OH-B[a]A and E_2_ on MCF-7 cells revealed the expression profiles of E-Cadherin, Vimentin, MMP2, and MMP9 (Figure 8). As shown in Figure 8A,B, quantitative analysis demonstrated that the relative expression levels of E-Cadherin were 1.00 in the control group, 1.49 in the 0.5 nM E_2_ group, 1.43 in the 5 nM E_2_ group, 1.70 in the 0.4 μM 3-OH-B[a]A group, and 1.37 in the 2 μM 3-OH-B[a]A group, respectively. Treatment with E_2_ at concentrations of 0.5 nM and 5 nM significantly increased E-Cadherin protein expression relative to the control, with the 0.5 nM E_2_ group exhibiting a more pronounced upregulatory effect. Similarly, exposure to 0.4 μM 3-OH-B[a]A resulted in a significant upregulation of E-Cadherin protein expression, whereas treatment with 2 μM of the compound did not elicit a statistically significant change. Collectively, these findings indicate that 3-OH-B[a]A at 0.4 μM enhances E-Cadherin expression in human breast cancer MCF-7 cells.

As illustrated in Figure 8C,D, quantitative analysis showed that the relative expression levels of Vimentin were 1.00 in the control group, 1.21 in the 0.5 nM E_2_ group, 1.27 in the 5 nM E_2_ group, 1.38 in the 0.4 μM 3-OH-B[a]A group, and 1.24 in the 2 μM 3-OH-B[a]A group, respectively. Treatment with 5 nM E_2_ resulted in a significant increase in Vimentin protein expression compared with the control. A comparable elevation in Vimentin levels was observed in MCF-7 cells exposed to 0.4 μM 3-OH-B[a]A, whereas treatment with 2 μM of the compound did not induce a statistically significant change. Collectively, these data indicate that 3-OH-B[a]A at 0.4 μM upregulates Vimentin expression in human breast cancer MCF-7 cells.

As shown in Figure 8E,F, quantitative analysis showed that the relative expression levels of MMP2 were 1.00 in the control group, 0.91 in the 0.5 nM E_2_ group, 1.28 in the 5 nM E_2_ group, 1.20 in the 0.4 μM 3-OH-B[a]A group, and 1.07 in the 2 μM 3-OH-B[a]A group, respectively. Exposure to 5 nM E_2_ significantly increased MMP2 protein expression relative to the control, whereas treatment with 0.5 nM E_2_ did not result in a statistically significant change. Similarly, MCF-7 cells treated with 0.4 μM 3-OH-B[a]A exhibited a marked upregulation of MMP2 expression, while the 2 μM concentration had no significant effect. Collectively, these findings indicate that exposure to 3-OH-B[a]A at 0.4 μM significantly enhances MMP2 protein expression in human breast cancer MCF-7 cells.

As shown in Figure 8G,H, quantitative analysis revealed that the relative expression levels of MMP9 were 1.00 in the control group, 1.46 in the 0.5 nM E_2_ group, 1.42 in the 5 nM E_2_ group, 1.47 in the 0.4 μM 3-OH-B[a]A group, and 1.30 in the 2 μM 3-OH-B[a]A group, respectively. Treatment with E_2_ at 0.5 nM and 5 nM significantly increased MMP9 protein expression compared with the control. Similarly, exposure of MCF-7 cells to 0.4 μM 3-OH-B[a]A resulted in a significant upregulation of MMP9 levels, whereas treatment with 2 μM of the compound did not induce a statistically significant change. Taken together, experimental data indicate that exposure to 3-OH-B[a]A at 0.4 µM significantly upregulates MMP9 protein expression in human breast cancer MCF-7 cells.

**Figure 9:**
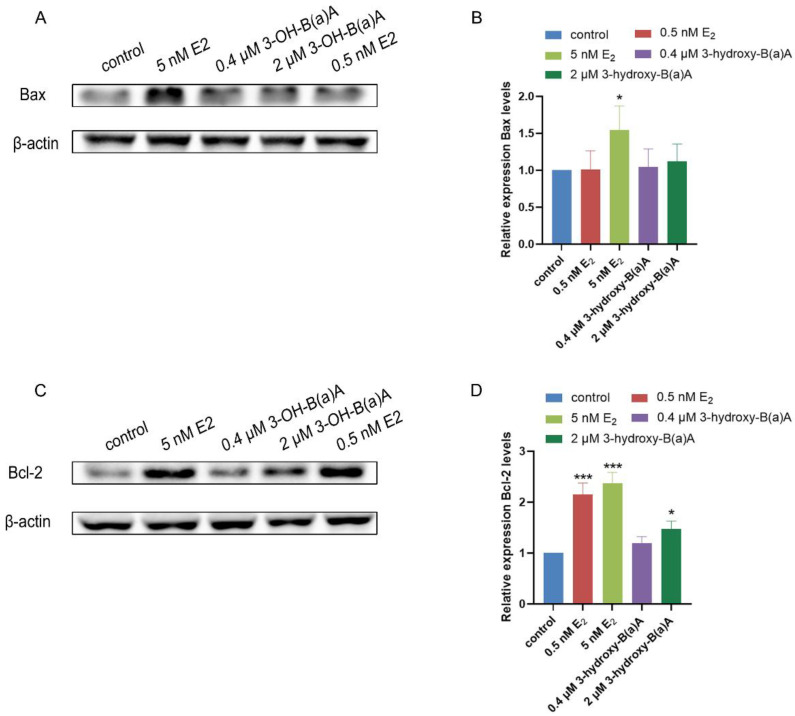
Effect of 3-OH-B[a]A and E_2_ on the expression of apoptosis-related proteins Bax and Bcl-2 in MCF-7 cells (*n* = 3). (**A**) Representative immunoblot showing Bax expression following treatment with 3-OH-B[a]A and E_2_. (**B**) Quantitative analysis of relative Bax protein expression corresponding to panel (**A**). (**C**) Representative immunoblot showing Bcl-2 expression. (**D**) Quantitative analysis of relative Bcl-2 protein expression corresponding to panel (**C**). Data are presented as mean ± SD. * *p* < 0.05 and *** *p* < 0.001 versus the vehicle control.

Figure 9 presents Western blot analyses examining the effects of 3-OH-B[a]A and E_2_ on the expression of the apoptosis-related proteins Bax and Bcl-2 in MCF-7 cells. As shown in Figure 9A,B, quantitative analysis showed that the relative expression levels of Bax were 1.00 in the control group, 1.01 in the 0.5 nM E_2_ group, 1.54 in the 5 nM E_2_ group, 1.05 in the 0.4 μM 3-OH-B[a]A group, and 1.12 in the 2 μM 3-OH-B[a]A group, respectively. Treatment with 5 nM E_2_ resulted in a significant upregulation of Bax protein expression relative to the control, whereas exposure to 0.5 nM E_2_ did not produce a statistically significant change. Among the 3-OH-B[a]A-treated groups, neither 0.4 μM nor 2 μM exposure resulted in a statistically significant change in Bax protein expression. Accordingly, under the present experimental conditions, 3-OH-B[a]A at these concentrations does not measurably affect Bax expression in MCF-7 cells.

As shown in Figure 9C,D, quantitative analysis demonstrated that the relative expression levels of Bcl-2 were 1.00 in the control group, 2.15 in the 0.5 nM E_2_ group, 2.37 in the 5 nM E_2_ group, 1.20 in the 0.4 μM 3-OH-B[a]A group, and 1.47 in the 2 μM 3-OH-B[a]A group, respectively. Treatment with E_2_ at concentrations of 0.5 nM and 5 nM significantly increased Bcl-2 protein expression relative to the control. Similarly, exposure to 2 μM 3-OH-B[a]A resulted in a marked upregulation of Bcl-2 levels, whereas treatment with 0.4 μM of the compound did not induce a statistically significant change. Collectively, these findings indicate that 3-OH-B[a]A at 2 μM enhances Bcl-2 protein expression in human breast cancer MCF-7 cells.

## 4. Discussion

This study systematically investigated the estrogenic activity of 3-OH-B[a]A in the estrogen receptor-positive breast cancer cell line MCF-7 and its influence on malignant cellular phenotypes. The findings demonstrate that 3-OH-B[a]A exhibits pronounced estrogen-like activity, leading to enhanced cell proliferation and migratory capacity, concomitant with a suppression of apoptotic responses in MCF-7 cells. Notably, this estrogenic activity did not translate into a significant alteration of the invasive capacity of MCF-7 cells. Previous studies have established that estrogenic compounds can drive breast cancer progression through multiple, interrelated molecular and cellular mechanisms. For example, environmental endocrine disruptors such as bisphenol A (BPA) and dodecafluoro-1,6-diiodohexane (PFHxDI) have been reported to significantly stimulate the proliferation of estrogen receptor-positive breast cancer cell lines, including MCF-7 and T47D [33,34]. Subsequent studies have demonstrated that BPA markedly enhances the migration and invasive capabilities of MCF-7 cells by suppressing KRT14 expression [35]. In addition, exposure to BPA and triclosan has been shown to induce anti-apoptotic effects in VM7Luc4E2 breast cancer cells [36]. Collectively, these findings suggest that exposure to estrogenic endocrine disruptors may contribute to an increased risk of breast cancer development and progression.

Although the in vitro concentrations used in this study are relatively higher than typical environmental levels, they were selected to elucidate the molecular mechanisms by which 3-OH-B[a]A exerts estrogenic effects and pro-tumorigenic potential. In real-world scenarios, humans are exposed to PAHs primarily through inhalation of contaminated air, ingestion of contaminated food and water, and dermal contact [37], with these compounds typically present at trace levels in environmental media such as water, food, and atmosphere [38]. Even at such low environmental concentrations, long-term cumulative exposure may still induce endocrine-disrupting effects and contribute to the development and progression of breast cancer [39,40]. Therefore, our findings provide mechanistic evidence for understanding the potential health risks of PAH metabolites under realistic exposure scenarios and highlight the importance of environmental monitoring and risk assessment for PAH pollutants.

Endocrine disruptors can modulate estrogenic signaling through both genomic and non-genomic pathways via multiple mechanisms, including direct interaction with estrogen receptors, indirect activation of transcriptional regulators such as the aryl hydrocarbon receptor, and disruption of enzymatic processes involved in estrogen biosynthesis and metabolism [41]. Among these mechanisms, non-genomic estrogenic signaling frequently involves the rapid activation of kinase-driven pathways. For example, the prototypical endocrine disruptor diethylstilbestrol (DES) has been shown to stimulate PI3K/AKT signaling rapidly [42]. Substantial evidence further indicates that aberrant activation of the PI3K/AKT pathway is a common feature of multiple cancer types, where it plays a critical role in driving tumor proliferation, survival, and metastatic progression [43,44]. This study confirmed that 3-OH-B[a]A stimulates MCF-7 cell proliferation, as demonstrated by EdU incorporation and colony formation assays (Figure 2B and Figure 3B). To further elucidate the underlying mechanism, the effects of 3-OH-B[a]A on the expression of AKT and its phosphorylated form, p-AKT, within the PI3K/AKT signaling pathway were investigated. The results demonstrated that treatment with 0.4 μM 3-OH-B[a]A significantly increased total AKT protein expression, while no corresponding change in p-AKT levels was observed. In contrast, exposure to 2 μM 3-OH-B[a]A did not produce significant changes in either AKT or p-AKT expression (Figure 7A–D). These findings suggest that the proliferative effects of 3-OH-B[a]A are unlikely to be mediated predominantly through rapid, non-genomic activation of the PI3K/AKT signaling pathway. Accordingly, the effect of 3-OH-B[a]A on the expression of c-Myc, a key transcription factor implicated in tumorigenesis and the regulation of cell cycle progression and cellular proliferation, was further investigated [45,46]. The results demonstrated that exposure to 0.4 μM 3-OH-B[a]A significantly increased c-Myc protein expression, suggesting that this compound may promote cell cycle progression and proliferation activity through c-Myc upregulation (Figure 7E,F). This effect represents a plausible mechanistic basis for the observed pro-proliferative phenotype.

Epithelial–mesenchymal transition (EMT) is a fundamental biological process that enables tumor cells to acquire migratory and invasive properties, thereby playing a key role in cancer metastasis [47,48]. In this study, wound healing assays demonstrated that 3-OH-B[a]A significantly enhances the migratory capacity of MCF-7 cells (Figure 4C–E). To explore the underlying mechanism, the effects of 3-OH-B[a]A on the expression of key EMT markers, including the epithelial marker E-Cadherin and the mesenchymal marker Vimentin, were further assessed. The results demonstrated that 3-OH-B[a]A concurrently upregulates the expression of both E-Cadherin and Vimentin in MCF-7 cells (Figure 8A–D). This apparent paradoxical finding may reflect the presence of intermediate, partial, or hybrid EMT states in tumor cells. Increasing evidence suggests that cells undergoing EMT can occupy transitional phenotypic states characterized by the concurrent expression of both epithelial and mesenchymal markers, with the capacity to dynamically interconvert between these states [49]. Consistent with this concept, transcriptomic analyses have identified mixed EMT phenotypes in models of skin squamous cell carcinoma and breast tumors [50]. Collectively, our findings suggest that 3-OH-B[a]A may drive MCF-7 cells toward a distinct intermediate EMT state, conferring enhanced migratory capacity while preserving partial epithelial features. This phenotype warrants further investigation in future studies using complementary functional and molecular assays to delineate the underlying regulatory mechanisms.

Local tumor invasion is a highly coordinated process involving cytoskeletal remodeling, dynamic regulation of cell–matrix adhesion, and degradation of the extracellular matrix (ECM) [51]. Matrix metalloproteinases (MMPs) play a central role in facilitating tumor cell infiltration and metastatic dissemination by proteolytically degrading ECM components [52]. Preliminary Transwell invasion assays indicated that 3-OH-B[a]A did not produce a significant change in the invasive capacity of MCF-7 cells (Figure 5B). However, subsequent mechanistic analyses revealed that this compound markedly upregulated the protein expression of the invasion-associated enzymes MMP2 and MMP9 (Figure 8E–H). The apparent discrepancy between phenotypic outcomes and molecular markers may be attributable to the intrinsically low invasive potential of MCF-7 cells [53]. Although MMP2 and MMP9 were upregulated, the manifestation of a fully invasive phenotype likely requires coordinated engagement of additional processes, including cell–matrix adhesion dynamics and associated signaling pathways [54]. Notably, it has also been observed that 3-OH-B[a]A upregulated the expression of the epithelial adhesion protein E-Cadherin. Given its critical role in maintaining intercellular adhesion [55], increased E-Cadherin expression may partially offset the pro-invasive effects associated with MMP2 and MMP9 upregulation, thereby limiting the emergence of a measurable increase in overall invasive capacity.

Apoptosis, the principal form of programmed cell death, serves a crucial regulatory function in preserving tissue homeostasis and facilitating the removal of damaged or aberrant cells [56]. Apoptotic signaling is predominantly governed by the B-cell lymphoma/leukemia 2 (BCL-2) protein family, which comprises both pro-apoptotic members, such as Bax, and anti-apoptotic members, including Bcl-2. The dynamic balance between these opposing regulators ultimately determines whether a cell survives or undergoes programmed cell death [57]. TUNEL assays performed in this study demonstrated that 3-OH-B[a]A suppresses apoptotic activity in MCF-7 cells (Figure 6B). To elucidate the underlying mechanism, its effects on the expression of the apoptosis-related proteins Bax and Bcl-2 were examined. The results revealed that treatment with 2 μM 3-OH-B[a]A significantly increased Bcl-2 protein expression, while Bax levels remained unchanged (Figure 9A–D). As a central anti-apoptotic regulator, overexpression of Bcl-2 enhances tumor cell resistance to programmed cell death, thereby promoting cell survival and proliferation and accelerating tumor progression [58]. Accordingly, these findings suggest that 3-OH-B[a]A may potentiate the anti-apoptotic capacity of MCF-7 cells through the upregulation of Bcl-2 expression, providing a mechanistic basis for its observed pro-proliferative effects.

## 5. Conclusions

This study demonstrates that 3-OH-B[a]A exhibits pronounced estrogen-like activity in vitro, modulating malignant phenotypes in breast cancer cells. Specifically, it significantly enhanced proliferation at concentrations of 0.4 μM and 2 μM, promoted migration at 0.4 μM, and suppressed apoptosis at 2 μM. Mechanistically, these effects are associated with coordinated modulation of estrogen-responsive and metastasis-related proteins, indicating activation of estrogenic signaling pathways that promote tumor-associated behaviors. Collectively, these findings identify 3-OH-B[a]A as a biologically active polycyclic aromatic hydrocarbon (PAH) derivative with the capacity to disrupt endocrine signaling and contribute to breast cancer-relevant cellular processes. These results provide mechanistic evidence supporting the potential role of PAH metabolites in hormone-dependent carcinogenesis and have important implications for environmental health risk assessment and preventive strategies targeting endocrine-disrupting pollutants. While this study is limited to in vitro models, further validation using in vivo systems, such as breast cancer xenograft models, will be essential to establish the estrogenic and tumor-promoting effects of 3-OH-B[a]A definitively in vivo.

## Data Availability

The original contributions presented in this study are included in the article. Further inquiries can be directed to the corresponding author.
